# Effect of Different Dietary Patterns on Macronutrient Composition in Human Breast Milk: A Systematic Review and Meta-Analysis

**DOI:** 10.3390/nu15030485

**Published:** 2023-01-17

**Authors:** Qinghua Xi, Weixin Liu, Tianyuan Zeng, Xuan Chen, Ting Luo, Zeyuan Deng

**Affiliations:** 1 State Key Laboratory of Food Science and Technology, Nanchang University, 235 East Nanjing Road, Nanchang 330047, China; 2 Jiangxi Province Key Laboratory of Preventive Medicine, Nanchang University, 461 Bayi Street, Nanchang 330006, China

**Keywords:** association analysis, human milk macronutrient, dietary patterns, quantitative reviews

## Abstract

The aim of this meta-analysis was to systematically review the relationships between lactation diet and the composition of mature breast milk through screening multiple databases and gray literatures, with priority given to quantitative articles published in Chinese and English. We identified 27 cross-sectional studies that included 4355 lactating women. According to these data, dietary patterns were categorized into four patterns based on the fat and protein energy supplying ratio, including rational-fat and rational-protein dietary (RR), high-fat and high-protein dietary (HH), rational-fat and high-protein dietary (RH), and high-fat and rational-protein dietary (HR). The results showed the fat content in mature milk was increased with increments of fat intake, while both the protein and lactose contents in mature milk did not increase with their intakes for lactating mothers. Among these four dietary patterns, the energy ratio of macronutrients in human milk at the RR was the closest to the estimated energy ratio for infants aged 0–6 M. In conclusion, our study represents that the rational dietary pattern should be advocated, and the irrational dietary patterns, especially high-fat and high-protein dietary patterns, should be avoided during lactation stage.

## 1. Introduction

Breast milk is the full value food in the first six months for infants, and the appropriate proportion of ingredients is easily absorbed by the baby’s gastrointestinal tract. Optimal nutrition for lactating mothers (LMs) is critical to both the mother’s health and the infant’s short-term and even long-term growth and development. The reasonable intakes of macronutrients, including protein, fat, and carbohydrates, are associated with a decreased incidence of infections and chronic diseases for infants, such as gastrointestinal infections, autoimmune diseases, and metabolic syndrome [1,2]. Some researchers found that the self-selected nutrient intake of LMs was lower than the recommended level, which might influence the nutritional composition of breast milk [3,4,5]. The high-quality protein in breast milk is an important substance, which has a positive relationship with body weight gain and body length of infants. Insufficient total energy, especially a lack of protein, can cause infant growth retardation, shorter stature, and destroyed immune activity [6,7]. Fatty acids are proven to be a critical component in development of the infant’s central nervous system. A lack of *n* − 3 polyunsaturated fatty acids could impact the intellectual development of children and increase the prevalence of brain development-related diseases [8,9,10]. Some studies reported the composition of Docosahexaenoic acid (DHA), eicosapentaenoic acid (EPA), arachidonic acid (ARA), and trans-fatty acids in human milk were also directly linked to maternal dietary intake [11,12,13]. Nowadays, the nutrient intake of LMs is influenced by the traditional habits and a westernized lifestyle shifting to high protein and/or high fat dietary patterns [14]. When the intake of protein intake exceeds the body’s needs, it can put people at higher risk of kidney and liver disorders and reduce their uptake of vitamins, minerals, fiber, and other nutrients [15,16,17]. When fat intake exceeds the body’s needs, it may place the maternal individual and their offspring at a higher risk of obesity, hypertension, and insulin dysfunction [18,19]. All these factors have increased the current prevalence of chronic diseases, including cardiovascular disease, diabetes mellitus, and several other diseases [5,15,20,21]. However, few studies systematically evaluate the effects of different dietary patterns on the composition of breast milk during the early stage of lactation.

There are many factors affecting the content of breast milk, including maternal factors (race, age, diet, and lactation stage), infant factors (birth order and gender), and physiological factors, among which the maternal diet is only one factor that can be interfered [5,22,23]. Some studies have shown that human milk macronutrients (protein, fat, and carbohydrates) were influenced by dietary habit in different regions [24,25,26,27]. One study reported that total protein content in milk was affected by body composition [28]. And other studies also showed that geographical location, lactation period, age, and pre-BMI value affected the protein content in human milk [29]. The fat, especially fatty acids, vitamins, and dry matter in breast milk were affected by the diet [24,30]. Some researchers found that the content of EPA in breast milk in Indian women was higher than that in Malaysian women, even though these people lived in the same area, and the protein content of breast milk in Thai mothers was higher than that in Japanese mothers [31,32]. Some findings further showed that protein and fat content of breast milk in rural areas were lower than those in urban areas [33,34]. Conversely, some studies have indicated no correlation between breast milk production and macronutrient intake. Therefore, the causal relationship between breast milk composition and maternal diet is still controversial [4,5,35].

In published reports, while cross-sectional studies were mainly used in investigating and analyzing the effect of dietary intake on the breast milk composition of LMs, its shortcomings were that they could not provide evidence to prove the temporal relationship among different variables [5,35,36]. The classic reviews, which involve systematic reviews, proposed that rational dietary components could improve nutritional status during lactation [5,15], but there was no comprehensive research on breast milk composition levels under different dietary patterns. Meta-analyses pooled and yielded quantitative evidence on a causal link that offered objective and quantified results from a carefully selected pool of the most homogeneous studies. Therefore, meta-analyses draw the relationship between the macronutrient composition of breast milk and dietary patterns.

This review aimed to explore how different maternal dietary patterns affected the macronutrients in breast milk by assessing the values and heterogeneous sources of breast milk macronutrients. A holistic perspective was used to assess the associations between the macronutrients in breast milk and dietary patterns. All the observational studies and interventional studies, classic reviews, and systematic reviews were pooled and reviewed in our study to achieve these objectives. Then, four dietary patterns were defined based on the energy ratios of protein and fat intake in total energy, and the associations between the macronutrients of breast milk and dietary patterns were finally drawn by meta-analyses.

## 2. Materials and Methods

The results of a systematic review were graphically summarized to show how breast milk composition changes with diet patterns. Since breast milk macronutrients varied greatly with lactation stages, only the values of macronutrients from 1–3 M of mature breast milk were collected for the meta-analyses to decrease the systematic error and obtain the accurate estimates.

### 2.1. Data Sources, Search Strategy, and Selection Criteria

This review was conducted in accordance with the Preferred Reporting Items for Systematic Review and Meta-Analysis (PRISMA) Guidelines [37]. The literature searches for both English and Chinese were published from 1 January 2000 to 31 December 2022. Titles and abstracts of all the articles were checked from the databases of Web of Science (https://www.webofscience.com/wos/alldb/basic-search, accessed on 23 June 2022), PubMed (https://pubmed.ncbi.nlm.nih.gov, accessed on 23 June 2022), the China National Knowledge Infrastructure (CNKI) (http://cnki.net, accessed on 23 June 2022), and WanFang (http://www.wanfangdata.com.cn/index.html, accessed on 23 June 2022). The following search strategy was applied: “(human OR breast) AND (milk) AND (dietary intake) AND (composition OR concentration OR content OR quantification OR amount)”. A snowball method, through manual cross-references from retrieved articles, was conducted to identify any additional relevant publications.

The inclusion and exclusion criteria are listed in the PICOS table (Table S1). Quality of included studies was assessed as previously described. After that, the full texts of all the remaining studies were carefully read to determine whether they met the set eligibility criteria or not. All the articles were examined for potential fit to the inclusion criteria by the two authors (Xi and Zeng). If there existed disagreement, the determination would be made by a third author (Liu) after fully discussing with the two authors.

### 2.2. Data Extraction and Processing

The following information was collected: (1) the first author, publication year, age, body mass index (BMI) of mothers, regions, and sample size of the study; (2) four dietary patterns; (3) the outcome index, mean values, and standard deviation (SD) of macronutrients in the breast milk of the LMs; and (4) the energy ratio of macronutrients in the total energy. Dietary energy mainly comes from protein, fat, and carbohydrates. According to the Chinese recommended reference values of energy-supplying LMs [38], a reasonable dietary pattern was defined as one in which the energy-supplying ratios from fat and protein accounted for 20–30% and 12–15% of the total energy, respectively. Thus, four dietary patterns were categorized as (1) The RR pattern referred to 20–30% and 12–15% of the energy supplied from fat and protein, respectively; (2) The HH pattern referred to more than 30% and over 15% of energy supplied from fat and protein, respectively; (3) The RH pattern referred to 20–30% and more than 15% of the energy supplied from fat and protein, respectively; (4) The HR pattern referred to more than 30% and 12–15% of energy supplied from fat and protein, respectively. The energy content of breast milk was calculated as the mass of each macronutrient multiplied by the corresponding coefficient of energy, where the contents of macronutrients were reported in grams per 100 mL and the coefficients of energy were used as 4 kcal/g, 9 kcal/g, and 4 kcal/g for protein, fat, and carbohydrates, respectively. Means and standard deviations (SDs) of macronutrients concentrations were extracted, and their units were all converted to g/100 g.

### 2.3. Quality Assessment

The quality of each included article was evaluated using the Agency for Health Care Research and Quality (AHRQ) scale, which is the cross-sectional study evaluation criteria. The scale contained 11 items; if each item was “yes”, one point would be obtained when each item was “yes”, while “zero” points would be obtained if it was “no” or “unclear”. In total, a score of 8–11 points indicated high quality, 4–7 points indicated moderate quality, and a score of 0–3 points indicated low quality [39].

### 2.4. Statistical Analysis

Protein, fat, and lactose in breast milk were selected as the evaluation indices. The mean differences, 95% confidence intervals (CI), and effect sizes of these macronutrient contents were calculated. Stata 20.0 software was used for meta-analysis, including heterogeneity testing, sensitivity analysis, meta-regression, subgroup analysis, and the evaluation of publication bias. A random-effects model was used to estimate the weighted mean. The statistical results of *I*^2^ was used to quantify the degree of heterogeneity, and a high degree of heterogeneity could be confirmed when the *I*^2^ value was greater than 75%. The Egger test was used to evaluate publication bias (when the *p* value was less than 0.1, publication bias existed). Revman 5.4 software was used to compare the macronutrient contents of breast milk in high-fat and rational-fat dietary patterns.

## 3. Results

### 3.1. The Characteristics of the Articles

**Figure 1:**
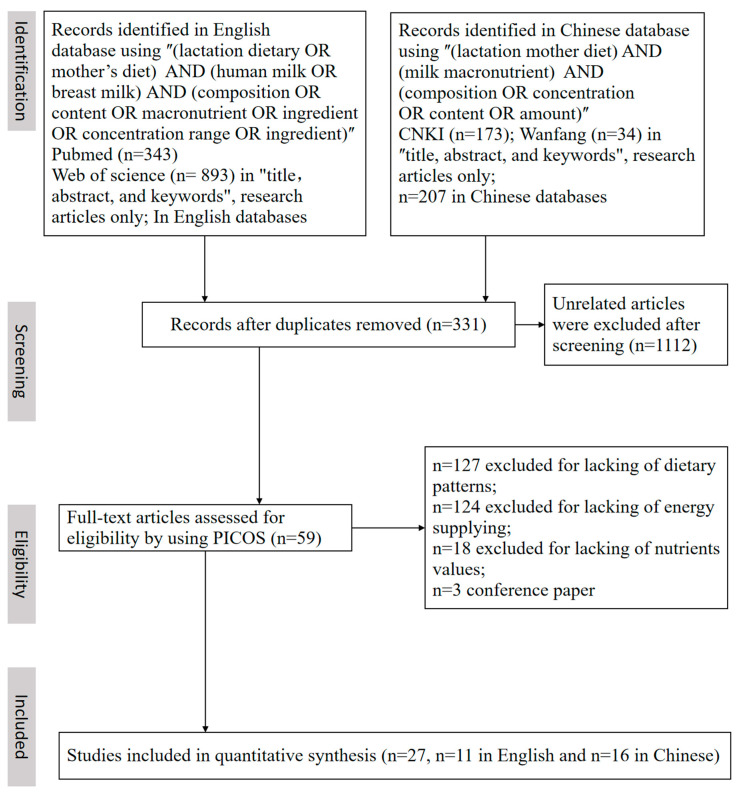
Flow chart of literature searching and screening. (PRISMA). The process of selecting the articles that meet the inclusion and exclusion criteria was listed in the PICOS table (Table S1).

After the database and article reference lists had been searched, 59 potentially relevant articles were retrieved. Twenty-seven articles met the inclusion criteria, and most of the studies were finally excluded for reasons provided in Figure 1. The study selection process and according to results are summarized in the flow diagram. These macronutrient-related values were extracted from a total of 27 documents, including 16 in Chinese and 11 in English, which contained 47 sets of data with a total of 4355 participants (aged 18–45 y with a BMI between 18.9 and 24.9). According to the above evaluation criterion, 26 articles were identified as high quality, and one article was identified as having moderate quality. The corresponding scores for the quality assessment are listed in Table 1. Based on our statistical standard, data from seven of the studies were categorized as the RR pattern, nineteen were categorized as the HH pattern, sixteen were categorized as the RH pattern, and five were categorized as the HR pattern. Among these studies, 25 of them contained all of the protein-, fat-, and lactose-related data, only one contained protein-related data, and the other one contained fat-related data. Seventeen of the studies used a breast milk analyzer, and ten of the studies used the classic manual method to analyze the macronutrient composition of breast milk. The breast milk analyzer was much easier and faster than the classic manual method in detecting breast milk components.

### 3.2. Meta-Analysis of Macronutrients

#### 3.2.1. Protein

**Figure 2:**
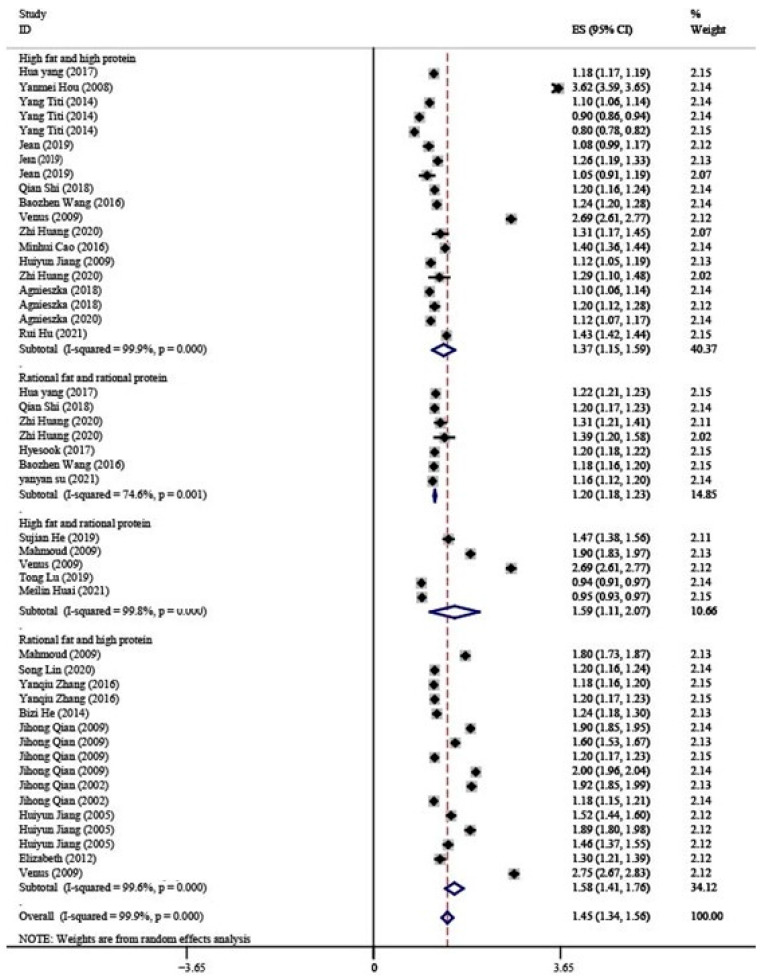
Forest map shows the mean values of protein contents in breast milk at four different dietary patterns [31,32,33,34,35,36,37,38,39,40,41,42,43,44,45,46,47,48,49,50,51,52,53,54,55,56,57,58,59,60,61].

The results of the mean values of protein content in breast milk at different dietary patterns are shown in Figure 2. The results of this study showed that the values of milk protein at HH, RR, HR, and RH patterns were 1.37 g/100 mL (95% CI: 1.15–1.59), 1.20 g/100 mL (95% CI: 1.18–1.23), 1.59 g/100 mL (95% CI: 1.11–2.07), and 1.58 g/100 mL (95% CI: 1.41–1.76), respectively. The lowest protein value was from the RR pattern, and the highest was from the HR protein pattern. It displayed that milk protein content increased with fat and protein intake at the same rational dietary pattern (HR > RR, RH > RR), but decreased with fat and protein intake at the same high dietary pattern (HH < RH, HH < HR).

#### 3.2.2. Fat

**Figure 3:**
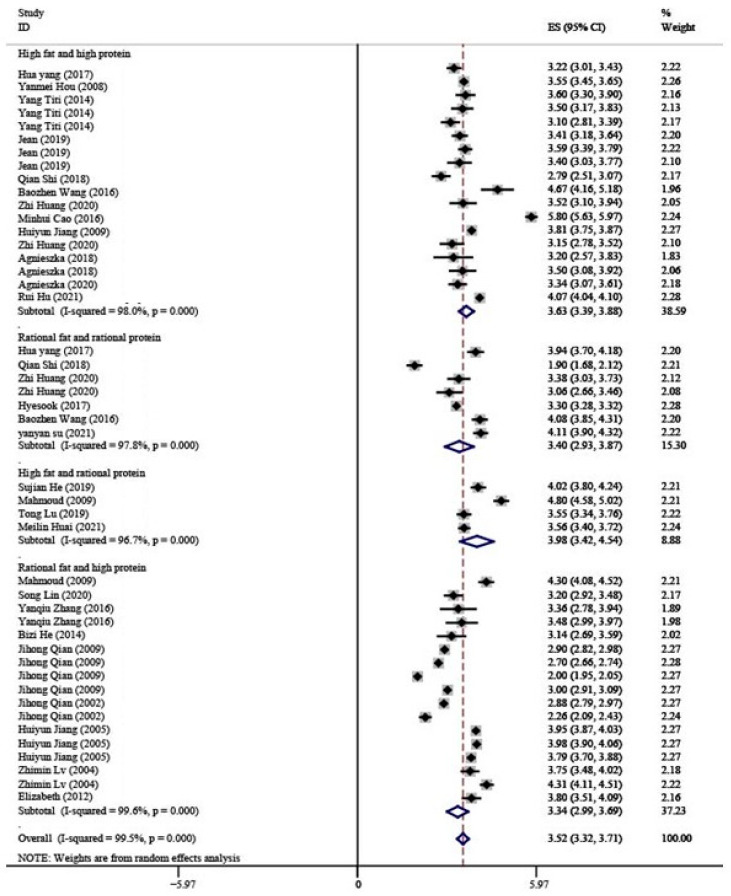
Forest map show the mean values of fat contents in breast milk at four different dietary patterns [26,27,28,29,30,31,32,33,34,35,36,37,38,39,40,41,42,43,44,45,46,47,48,49,50,51,52,53,54,55,56,57,58,59,60,61].

The results of the mean values fat contents in breast milk at different dietary patterns are shown in Figure 3. The Fat values for HH, RR, HR, and RH patterns were 3.63 g/100 mL (95% CI: 3.39–3.88), 3.40 g/100 mL (95% CI: 2.93–3.87), 3.98 g/100 mL (95% CI: 3.42–4.54), and 3.34 g/100 mL (95% CI: 2.99–3.69), respectively. The mean fat values at the high-fat pattern were higher than those at the rational-fat pattern, which showed that the fat content in breast milk changed with maternal fat intake at the same protein level (HH > RH, HR > RR).

#### 3.2.3. Lactose

**Figure 4:**
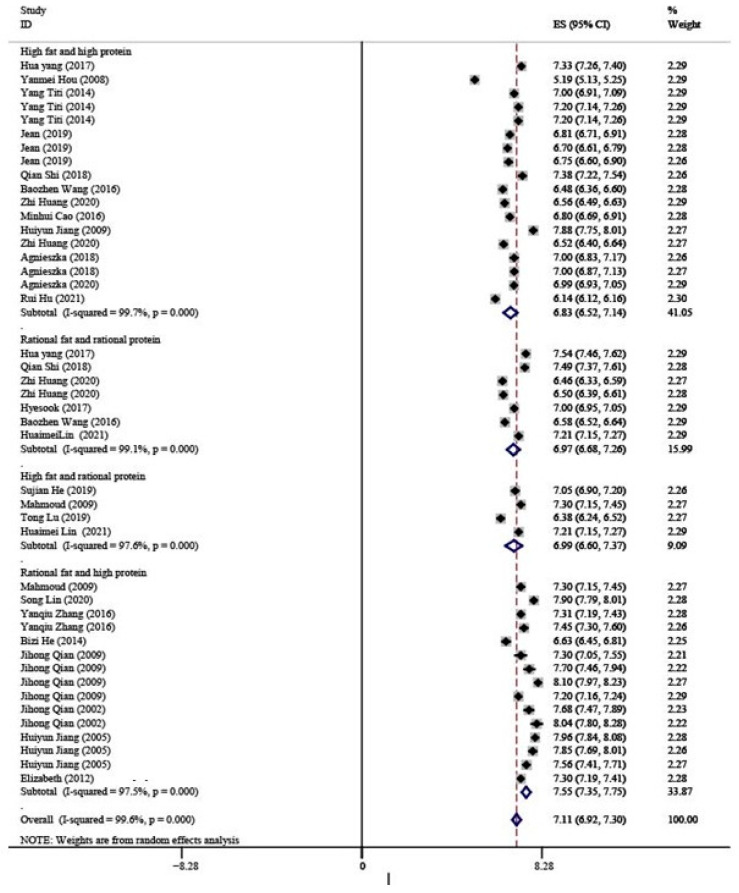
Forest map shows the mean values of lactose contents in breast milk at four different dietary patterns [26,27,28,29,30,31,32,33,34,35,36,37,38,39,40,41,42,43,44,45,46,47,48,49,50,51,52,53,54,55,56,57,58,59,60,61].

The results of the mean values of lactose contents in breast milk at different dietary patterns are shown in Figure 4. Lactose values at HH, RR, HR, and RH patterns were 6.83 g/100 mL (95% CI: 6.52–7.14), 6.97 g/100 mL (95% CI: 6.68–7.26), 6.99 g/100 mL (95% CI: 6.60–7.37), and 7.55 g/100 mL (95% CI: 7.35–7.75), respectively. The highest value of lactose was from the RH pattern (7.55 g/100 mL), not from the HH pattern (HH 6.83 g/100 mL). Moreover, the lactose content increased with maternal fat and protein intake at the rational dietary level (HR > RR, RH > RR), but decreased with maternal fat and protein intake at the high fat and high protein dietary level (HH < RH, HH < HR).

#### 3.2.4. Energy Supply

The results of energy supply of macronutrients are shown in Table 2. At these four patterns (HH, RR, HR, and RH) of LMs, the percentages of the protein energy to the total energy in breast milk were 8.37%, 7.58%, 9.11%, and 9.49%, respectively; the fat energy to the total energy in breast milk were 49.90%, 48.36%, 51.30%, and 45.15%, respectively; and the lactose energy to the total energy in breast milks were 41.73%, 44.06%, 39.60%, and 45.35%, respectively. The energy ratios among protein: fat: lactose at these four patterns (HH, RR, HR, and RH) were 1: 5.96: 4.96, 1: 6.38: 5.81, 1: 5.63: 4.39, and 1: 4.76: 4.78, respectively. According to statistical energy percentage of macronutrients (protein = 7.14%, fat = 48.00%, lactose = 44.76%) in breast milk for 0–6 M-old infants [38], the energy of protein: fat: lactose ratio was 1:6.72:6.27. Therefore, RR is the most reasonable dietary pattern, which is closest to adequate intake of protein/fat/lactose in breast milk for 0–6 M-old infants.

Nevertheless, the results showed most *I*^2^ values were higher than 75% (*p* < 0.10), which indicated there was a high heterogeneity. Next, subgroup analyses and meta regression were carried out to explore the source of heterogeneity.

### 3.3. Subgroup Analysis

Four variables, including carbohydrate intake, total energy, region, and analytical method, were set as covariates to further identify the sources of heterogeneity. The total energy variable was firstly excluded from further analysis because it could not explain any resources of the heterogeneity. The other results indicated significant differences in *I*^2^ values at levels of carbohydrate intake, analytical method, and region.

#### 3.3.1. Carbohydrates

As depicted in Table 3, subgroup analysis results indicated the carbohydrate content in LMs’ diets significantly influenced the heterogeneity of breast milk content. A reasonable dietary pattern was defined as one in which carbohydrate energy-supplying ratios accounted for 50–60% of total energy. At the HH pattern, the *I*^2^ value of protein content in the high-carbohydrate subgroup decreased from 99.9% to 49.6%. While it decreased from 98.0% to 78.1%, and to 0.0%, respectively, in the rational-carbohydrate and low-carbohydrate subgroups. In the low-carbohydrate subgroup at the HR pattern, the *I*^2^ value of lactose decreased from 97.6% to 81.2%. The results of other subgroups showed no significant difference.

#### 3.3.2. Analytical Method

Based on our results presented in Table 4, subgroup analysis results displaying the manual analytical method significantly influenced the heterogeneity of breast milk content. At the HH and the RH dietary patterns, the *I*^2^ value of protein content decreased from 99.9% to 85.1% and from 99.6% to 24.2%, respectively. At the HH and RH dietary patterns, the *I*^2^ value of fat content decreased from 98.0% to 0.0% and from 99.6% to 0.0%, respectively, and at the HR dietary pattern, the *I*^2^ value of lactose decreased from 99.7% to 14.4%. The results of other subgroups showed no significant difference.

#### 3.3.3. Region

Subgroup analysis results showed that the different regions significantly influenced the heterogeneity of breast milk content as described in Table 5. In the West subgroup, at the HH pattern, the *I*^2^ value of protein content decreased from 99.0% to 85.2%, and in the North and South subgroups at the RR pattern, the *I*^2^ value of protein content decreased from 74.8% to 0.0%, respectively. In the West and EU subgroups of the HR dietary pattern, the *I*^2^ values of protein content decreased from 99.8% to 85.4% and 85.1%, respectively. In the North subgroup of the RH dietary pattern, the *I*^2^ values of protein content and fat content decreased from 99.6% to 46.2% and 88.5%, respectively. In the EU subgroup of HH and RR dietary patterns, the *I*^2^ value of lactose content decreased from 99.7% to 14.4%, and 99.1% to 14.4%, respectively. These differences might be caused by dietary culture and habits in different regions.

### 3.4. Meta-Regression

The results of meta-regression, as presented in Table S2, demonstrated that the carbohydrate-intake level and the region could explain some sources of protein heterogeneity at RH pattern (*p* < 0.01, respectively).

### 3.5. Publication Bias

Egger’s linear regression test showed that there was no significant publication bias (*p* > 0.05), except the *p* value of the protein item at the RH dietary pattern (*p* < 0.05). Trim and fill analysis also showed that the effect of publication bias was small, indicating that the results above were highly reliable.

### 3.6. Comparison of Macronutrient Content between High-Fat and Rational-Fat Patterns

**Figure 5:**
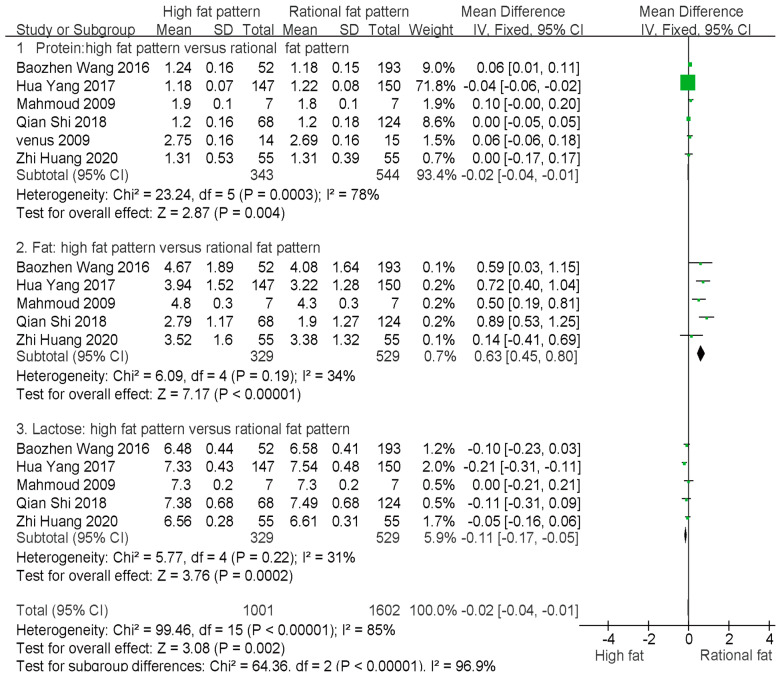
Forest map shows the comparison of breast milk macronutrient contents between high-fat dietary pattern and rational-fat dietary pattern [31,34,40,41,44,56].

The forest map is shown in Figure 5. The results showed the fat content in breast milk was higher (*p* < 0.05) at the high-fat pattern than that at the rational-fat pattern (SMD = 0.63, 95% CI = 0.45–0.80, *I*^2^ = 34%, fixed-effects model), but the protein and lactose contents were lower (*p* < 0.05) at the high-fat pattern than at the rational-fat pattern (SMD = −0.02, 95% CI = −0.04–0.01, *I*^2^ = 78%, random-effects model; SMD = −0.11, 95% CI = −0.17–0.05, *I*^2^ = 31%, fixed-effects model).

## 4. Discussion

The purpose of this meta-analysis was to explore the relationship between different dietary patterns and the macronutrient composition of breast milk within the first 1–3 M. Many researchers have investigated the nutrient contents of breast milk in different stages (colostrum, transitional milk, or mature milk). However, most previous narrative studies focused on a simple dietary influence on selected breast-milk composition, and they did not systematically assess the relationship between diet patterns and breast milk composition based on energy-supplying ratios [62,63,64]. The previous studies were compromised by their study design methodology, relatively small sample sizes, and/or statistical methods. As a result, they were probably limited by the diverse classification of dietary patterns such as principal component analysis and food source classification, and their lower statistical power may have been due to the small sample sizes [65,66,67]. Therefore, inconsistent or null results may occur even if there were an association between dietary patterns and milk composition. This meta-analysis divided the dietary patterns of LMs into four patterns according to the reference ratio of protein and fat energy to the total dietary energy, in order to search for the credible value range of macronutrients in breast milk and to establish the numerical relationship between LMs’ dietary intake and breast milk macronutrients by combining high-quality studies.

The present study demonstrates that breast milk components, especially fat content, are affected by different dietary patterns. Compared with the other macronutrients, fat is the most variable component of human milk, which is consistent with results of the previous study [68]. The fat in breast milk increased with the dietary fat intake of the LMs, while the contents of protein and lactose in breast milk fluctuated with different dietary patterns in this study. An observational study in Greek also showed a positive correlation between fat, especially polyunsaturated fatty acids (PUFAs), in breast milk and maternal fat intake [69]. Furthermore, our results show that the protein content of breast milk did not increase with the protein intake, which is consistent with reports of Mohammad’s research [56]. In contrast, the protein content in breast milk was negatively correlated with fat intake at the high-fat and high-protein patterns but positively correlated with fat intake at the RP pattern. Some experts believe that evaluating the association between dietary protein and protein content of breast milk should exclude the contribution of endogenous catabolic processes in the situation of an adequate protein–energy balance [70,71]. Moreover, one research study showed that lactose has no association with carbohydrate intake [72]. Another study showed that carbohydrates were the only macronutrients whose composition was significantly affected by the infant factors [26]. These research results mentioned the lactose content in breast milk was negatively correlated with fat intake at the high-fat and high-protein dietary patterns, which was consistent with the research of Wang et al. (2016) [40]. Some research showed that the source of energy in breast milk for toddler children is mainly from fat substances, while carbohydrates play a greater role in infant nutrition in the early stages. This could explain why in the early stages carbohydrate content was relatively stable to provide adequate energy [73]. Some researchers believed the nutrients in the breast milk would not be affected by deficient diet intake in the short term since stored nutrients in the mother’s body could be used, but that would affect excessive weight loss of the nursing mother in the long term [1,21,27]. Additionally, our research also shows the regions affected by the protein content in breast milk, which is also consistent with Chen et al.’s (2022) research [7].

On the other hand, we might judge whether the diet pattern of LMs is rational (RR) or irrational (high protein or high fat) through detecting and comparing macronutrient mean values in breast milk with the estimated mean values since the energy ratios of macronutrients (protein, fat, and lactose) in breast milk in a reasonable dietary pattern were closest to the estimated energy ratio of macronutrients in infants aged 0–6 M. The results of this study provide new objective evidence to guide the rational diet of LMs, which could further guide nutritional interventions if the LMs’ diet pattern was irrational. The American Heart Association (AHA) also did not recommend the high-protein and/or high fat-pattern since this probably limits the effectiveness of the body’s absorption of essential nutrients [74]. Thus, it is still a testable hypothesis that macronutrient metabolic mechanisms among different diet patterns should be considered in future studies.

## 5. Limitations

However, these findings should be interpreted with caution because of their limitations. Firstly, in addition to dietary patterns, many external factors influence the components of breast milk. Therefore, the different contributions of other influencing factors should also be considered though significant heterogeneity exists among the included studies. Although the carbohydrate intake level, region, and analyzer methods can explain parts of the heterogeneity, the residual heterogeneity needs to be explored and evaluated further in the future. Furthermore, because the composition of breast milk is a dynamic process to meet the accelerating growth of infants, the studies we included were cross-sectional studies in nature, analyzing only 1–3 M of mature milk but excluding colostrum and transitional milk. Most researchers have reported that protein content decreases with prolonged lactation, whereas the fat and lactose contents peak and then decrease with prolonged lactation [2,14,17,18]. Some previous researchers believed there is a less causal relationship between diet and breast milk composition in the very early stage [44,48]. What is more, another limitation is that we did not evaluate the dynamic process of breast milk. In the future, more cohort studies should be used to explore the comprehensive effects of multiple factors on the dynamic changes of breast milk composition based on the research variables such as race, region, maternal and infant BMI index, gestational age, and lactation stages. With the development of the economy, the Chinese dietary structure has changed to a high-fat, high-protein, and low-fiber intake, which often contains more protein and fat than the dietary recommendations for LMs due to adequate concerns about the health of lactating mothers and their nursing babies. The nutritional status of breastfeeding mothers usually varies with local customs in different regions. In this study, compared with the “Reference Intake of Dietary Macronutrients for Chinese Nursing Mothers (DRIS)”, most diet styles adopted by LMs belong to the high-protein pattern in the first 3 M lactation period because of the traditional nursing for LMs. However, the high protein pattern may not be conducive to the improvement of breast milk quality but would increase burden on the kidneys. Furthermore, fat, especially polyunsaturated fatty acids (PUFAs), is closely related to the development of the nervous system, but the high-fat content of breast milk may cause infant diarrhea during the neonatal period. Therefore, the consumption of fat probably causes high fat levels in breast milk and LM obesity. Infants taking high-fat breast milk are prone to indigestion and obesity, which may increase the risk for hypertension and coronary heart disease in adulthood [6,7,8].

Additionally, only the relationships between LMs’ diet and macronutrients in breast milk were investigated in this study. In fact, amino acid patterns, fatty acid composition, and different oligosaccharides in breast milk will also have vital impacts on infant development. Therefore, the effect of the LMs’ diet on these elements in breast milk should be considered in future research.

## 6. Conclusions

This meta-analysis provided a valuable range for breast milk composition at different dietary patterns, and it provided an objective basis for guiding a reasonable diet and avoiding high-protein and/or high-fat patterns during lactation. By detecting the contents of macronutrients in breast milk, we could propose the appropriate dietary intake level of LMs, which may guide the lactation diet scientifically. The results recommend taking a reasonable diet and avoiding a high-fat diet during lactation.

## Figures and Tables

**Table 1:** Characteristics of the included studies in different sample populations.

First Author, Year of Publication	Region	BMI (Mean ± SD)	Age (Mean ± SD)	Sample Size	Dietary Pattern	Breast Milk Composition /Method Analysis	Quality Score
Baozhen Wang 2016 [40]	Yinchuan①	--	--	193	RR①	①②③/①	11
	Yinchuan①	--	--	52	HH②	①②③/①	11
Qian Shi 2018 [41]	Beijing②	--	--	68	HH②	①②③/①	11
	Beijing②	--	--	124	RR①	①②③/①	11
Sujian He 2019 [42]	Guangzhou③	18.7 ± 2.5	27.5 ± 4.4	50	HR④	①②③/①	10
Tong Lu 2019 [43]	Changchun②	--	--	138	HR④	①②③/①	8
Zhi Huang 2020 [44]	Changsha③	--	18–40	55	HH②	①②③/①	10
	Changsha③	--	18–40	55	RR①	①②③/①	10
	Changsha③	--	18–40	55	RR①	①②③/①	10
Yang Hua 2017 [34]	Xian①	--	--	147	HH②	①②③/①	10
	Xian①	--	--	150	RR①	①②③/①	10
Yanmei Hou 2008 [45]	Jinan②	27.15 ± 4.3	22–35	240	HH②	①②③/①	7
Yang Titi 2014 [46]	Beijing②	23.3 ± 3.1	27.2 ± 3.9	85	HH②	①②③/①	9
	Beijing②	23.3 ± 3.1	27.2 ± 3.9	88	HH②	①②③/①	9
	Beijing②	23.3 ± 3.1	27.2 ± 3.9	90	HH②	①②③/①	9
Zhimin Lv 2004 [47]	Qingdao②	--	--	30	RH③	②/②	11
	Qingdao②	--	--	24	RH③	②/②	11
Jihong Qian 2009 [33]	Yangpu③	--	26–32	30	RH③	①②③/②	11
	Hongkou③	--	25–28	30	RH③	①②③/②	11
	Jingan③	--	26–29	30	RH③	①②③/②	11
Jihong Qian 2002 [48]	Shanghai③	--	22–36	90	RH③	①②③/②	11
	Shanghai③	--	22–36	30	RH③	①②③/②	10
Huiyun Jiang 2005 [49]	Nanning③	--	20–35	120	RH③	①②③/②	10
	Nanning③	--	20–35	120	RH③	①②③/②	10
	Nanning③	--	20–35	120	RH③	①②③/②	10
Huiyun Jiang 2009 [50]	Nanning③	-	20–35	120	HH②	①②③/②	10
Yanqiu Zhang 2016 [51]	Jinan②	20.8 ± 2.8	29.4 ± 2.6	33	RH③	①②③/①	10
Song Lin 2020 [52]	Shanghai③	20.8 ± 4.1	29.0 ± 3.9	112	RH③	①②③/①	8
Bizi He 2014 [53]	Beijing②	23.3 ± 2.8	31.7 ± 3.8	31	RH③	①②③/①	9
Jean 2019 [54]	Nancy④	23.2 ± 13.3	19–42	68	HH②	①②③/②	10
	Nancy④	23.2 ± 13.3	19–42	91	HH②	①②③/②	10
	Nancy④	23.2 ± 13.3	19–42	31	HH②	①②③/②	10
Hyesook 2017 [55]	Seoul②	22.1 ± 3.1	31.6 ± 3.2	255	RR①	①②③/②	10
Mahmoud 2009 [56]	TX④	23.1 ± 0.5	29.3 ± 1.0	7	RH③	①②③/②	9
	TX④	23.1 ± 0.5	29.3 ± 1.0	7	HR④	①②③/②	9
Venas 2009 [31]	Bangkok③	21.1 ± 0.75	26 ± 1.4	14	HR④	①/②	9
	Yokkaichi③	21 ± 0.65	30.5 ± 1.3	15	RH③	①/②	9
Elizabeth 2012 [57]	Cebu③	20.4 ± 3.5	23.8 ± 0.3	102	RH③	①②③/②	11
Minhui Cao 2016 [58]	Shanxi①	--	18–45	722	HH②	①②③/①	9
Agnieszka 2018 [28]	Poland④	23.0 ± 3.6	18–45	22	HH②	①②③/①	9
Agnieszka 2020 [26]	Poland④	20.4 ± 3.5	18–45	77	HH②	①②③/①	9
Rui Hu 2021 [59]	Shanghai③	29.3 ± 3.4	--	122	HH②	①②③/①	11
Yanyan Su 2021 [60]	Lanzhou②	--	21–40	102	RR①	①②③/①	11
Huaimei Lin 2021 [61]	Guangdong③	--	23–35	210	HR④	①②③/①	11

Region ① Areas adjacent to West of China ② Areas adjacent to North of China ③ Areas adjacent to South of China ④ Europe and US. Dietary pattern ① Rational fat and rational protein ② High fat and high protein ③ Rational fat and high protein ④ High fat and rational protein. Breast milk composition ① protein ② fat ③ lactose. Method analysis ① Breast Milk Analyzer ② manual classic method.

**Table 2:** Results of analysis of breast milk composition at different dietary patterns.

Dietary Pattern	Composition Index of Breast Milk	Mean (95% CI)	Heterogeneity Test		Egger’s (*p*)	Energy Supply (%)
			*I*^2^ (%)	*p*		
HH	Protein	1.37 (1.15–1.59)	99.9	0.00	0.64	8.37
	Fat	3.63 (3.39–3.88)	98.0	0.00	0.82	49.90
	Lactose	6.83 (6.52–7.14)	99.7	0.00	0.44	41.73
RR	Protein	1.20 (1.18–1.23)	74.6	0.00	0.78	7.58
	Fat	3.40 (2.93–3.87)	97.8	0.00	0.88	48.36
	Lactose	6.97 (6.68–7.26)	99.1	0.00	0.93	44.06
HR	Protein	1.59 (1.11–2.07)	99.8	0.00	0.15	9.07
	Fat	3.98 (3.42–4.54)	96.7	0.00	0.21	51.07
	Lactose	6.99 (6.60–7.37)	97.6	0.00	0.34	39.86
RH	Protein	1.58 (1.41–1.76)	99.6	0.00	0.03 *	9.49
	Fat	3.34 (2.99–3.69)	99.6	0.00	0.09	45.15
	Lactose	7.55 (7.35–7.75)	97.5	0.00	0.05	45.35

* Protein/Fat/Lactose: g/100 g.

**Table 3:** Values of subgroup with the carbohydrates.

Patterns	Protein Heterogeneity *I*^2^ Value (%)				Fat Heterogeneity *I*^2^ Value (%)				Lactose Heterogeneity *I*^2^ Value (%)			
	Group	LCD	RCD	HCD	Group	LCD	RCD	HCD	Group	LCD	RCD	HCD
HH	99.9	99.4	99.9	49.6 *	98.0	78.1 *	0.0 *	97.5	99.7	96.8	99.7	98.7
RR	74.8	--	74.8	--	97.8	--	97.8	--	99.1	--	99.1	--
HR	99.8	98.0	99.9	--	96.7	95.8	--	--	97.6	81.2 *	--	--
RH	99.6	--	99.4	99.8	99.6	--	99.4	98.3	97.5	--	97.2	97.6

LCD: Low carbohydrate dietary. RCD: Rational carbohydrate dietary. HCD: High carbohydrate dietary. * Significant decline of *I*^2^ Value (%) in subgroup.

**Table 4:** Values of subgroup with the analytical method.

Patterns	Protein Heterogeneity *I*^2^ Value (%)			Fat Heterogeneity *I*^2^ Value (%)			Lactose Heterogeneity *I*^2^ Value (%)		
	Group	Analyzer	Manual	Group	Analyzer	Manual	Group	Analyzer	Manual
HH	99.9	99.9	85.1 *	98.0	90.3	0.0 *	99.7	98.8	14.4 *
RR	74.8	72.3	--	97.8	97.8	--	99.1	99.1	--
HR	99.8	99.1	99.8	96.7	96.9	--	97.6	97.7	--
RH	99.6	24.2 *	99.6	99.6	99.7	0.0 *	97.5	98.0	97.5

* Significant decline of *I*^2^ Value (%) in subgroup.

**Table 5:** Values of subgroup with the region.

Patterns	Protein Heterogeneity *I*^2^ Value (%)					Fat Heterogeneity *I*^2^ Value (%)					Lactose Heterogeneity *I*^2^ Value (%)				
	Group	W	N	S	EU	Group	W	N	S	EU	Group	W	N	S	EU
HH	99.9	85.2 *	99.9	--	97.2	98.0	97.2	83.6	--	99.3	99.7	99.3	99.9	--	14.4 *
RR	74.8	90.0	0.0 *	0.0 *	--	97.8	94.5	99.7	99.9	--	99.1	99.1	99.2	--	14.4 *
HR	99.8	85.4 *	100	--	85.1 *	96.7	--	90.7	99.8	--	97.6	--	--	--	--
RH	99.6	--	46.2 *	99.7	--	99.6	--	88.5 *	99.7	--	97.5	--	--	96.2	97.9

W: Areas adjacent to West of China. N: Areas adjacent to North of China. S: Areas adjacent to South of China. EU: Europe and US. * Significant decline of *I*^2^ Value (%) in subgroup.

## Data Availability

Not applicable.

## Supplementary Materials

The following supporting information can be downloaded at: https://www.mdpi.com/article/10.3390/nu15030485/s1. Table S1. Inclusion criteria and exclusion criteria of article selection (PICOS). Table S2. Meta regression values of human breast milk (HM) contents with the three variables.
