# Supplementary material for: Effect of Different Dietary Patterns on Macronutrient Composition in Human Breast Milk: A Systematic Review and Meta-Analysis
Source: Nutrients. 2023 Jan 17;15(3):485. doi: 10.3390/nu15030485 (PMC9919556; doi:10.3390/nu15030485)
Supplement: Supplementary file 1 [file nutrients-15-00485-s001.zip › nutrients-2077548-supplementary.pdf]

Supplyment 1. Inclusion criteria and exclusion criteria of article selection (PICOS)

Parametar	Inclusion criteria	Exclusion criteria
Population	Healthy lactation mothers of healthy term infants	The studies populations had specific diseases
Intervention	N/A	N/A
Comparator	N/A	N/A
Outcomes	Nutritional status(Macronutrients intake in varies dietary patterns); Measured Macronutrients concentrations in breast milk(Mean values ± Standard Deviation)	the studies populations had specific diseases; the studies were not related to dietary patterns or breast milk composition; the studies data on energy supplying were not provided;
Study design	Crossing -sectional-study,RCTS	Abstracts,articles without full texts

Supplyment 2. Meta regression values of human breastmilk (HM) contents with the three variables

Diatery pattern	Variable name	Protein		Fat		Lactose	
		P	95% CI	P	95% CI	P	95% CI
HH	①	.54	[-.65,1.13]	.44	[-.35.71]	.44	[-0.94,0.45]
	②	.77	[-3.39,2.63]	.49	[-1.26,2.35]	.88	[-2.19,2.52]
	③	.81	[-1.10,1.36]	.58	[-.92,.56]	.67	[-1.15,0.78]
RR	①	--	--	--	--	--	--
	②	.42	[-.18,.09]	.91	[-3.77,3.49]	.89	[-1.79,1.96]
	③	.17	[-.04,.14]	.33	[-2.29,1.06]	.36	[-1.15,0.57]
RH	①	.00*	[-.48,1.04]	.25	[-.61,1.97]	.65	[-1.08,1.65]
	②	.79	[-.36,0.45]	.96	[-1.54,1.48]	.74	[-1.26,0.93]
	③	.00*	[.29,1.00]	.66	[-1.17,1.72]	.39	[-0.66,1.54]

Variable name *① Carbohydrate ② Analytical method ③ Region
